# Colloidal Dispersions of Gelled Lipid Nanoparticles (GLN): Concept and Potential Applications

**DOI:** 10.3390/gels3030033

**Published:** 2017-09-10

**Authors:** Mariana Carrancá Palomo, Victoria Martín Prieto, Plamen Kirilov

**Affiliations:** Université de Lyon (UCBL), Biologie Tissulaire et Ingénierie Thérapeutique UMR 5305–Vecteurs Colloïdaux et Transport Tissulaire, Institut des Sciences Pharmaceutiques et Biologiques, 8 Avenue Rockefeller, 69373 Lyon Cedex 08, France; mariana.carranca@ibcp.fr (C.P.M.); victoriabcn91@gmail.com (M.P.V.)

**Keywords:** gelled lipid nanoparticles (GLN), colloid dispersions, organogels, low molecular-mass organic gelator (LMOG), phase transition parameters, UV protection, drug delivery

## Abstract

The interest in using colloidal dispersions of gelled lipid nanoparticles (GLN) for different fields of application has increased in recent years, notably in cosmetic, dermatology, and/or pharmaceutics due to their capacity to immobilize compounds with poor water solubility. The pharmaceutical field desires to achieve lipophilic drug formulations which are able to conserve their stability, although it is well-known that emulsions and solid lipid nanoparticles (SLN) present a lack of stability over time, leading to system destabilization. Furthermore, stable colloidal dispersions of gelled oil particles do not affect the properties of the molecule to be delivered, and they result as an alternative for the previously appointed systems. This review is an attempt to present the reader with an overview of colloidal dispersions of GLN, their concept, formulation methods, as well as the techniques used for their characterization. Moreover, various application fields of organogel dispersions have been illustrated to demonstrate the potential application range of these recent materials.

## 1. Introduction

Deficient bioavailability due to certain physicochemical properties of some active substances is still a challenge in pharmaceutical science. Thus, new formulation strategies are required to overcome this problem—for example for poorly water-soluble compounds [1].

In the last years, the study of formulations to deliver water-insoluble molecules has increased. Emulsions and solid lipid nanoparticles (SLN) are up to now the most studied systems to deliver this type of molecule, and are unstable and have limited loading capacity. Therefore, there is still a demand for readily stable hydrophobic carriers with a higher loading capacity. Colloidal dispersions of gelled lipid nanoparticles (GLN) present an attractive alternative to surpass these limitations, since they may act as hydrophobic reservoirs [2,3].

Organogels are semi-solid systems (soft materials) in which a three-dimensional network composed of cross-linked gelator fibers immobilizes an organic liquid [4]. Organogels are principally classified based on the type of organogelator used, but also based on the nature of the organic liquid and its intermolecular interactions [5,6]. These organogelators can be divided into polymeric organic gelators (POG) and low molecular-mass organic gelators (LMOG) [5]. POG are high molecular-mass molecules which are capable of jellifying organic solvents, forming physical crosslinking points. These crosslinking points can be due to the formation of hydrogen bonds (inducing the formation of α-helices and β-sheets) for polypeptide polymers or π−π stacking interactions for p-conjugated polymers. LMOG are relatively small organic molecules (molecular weight < 2000 g/mol, usually even smaller than 500 g/mol) which are capable of forming a three-dimensional network around the organic liquid, thus gelling the organic liquid. LMOG have several advantages over POG: better biocompatibility, lower toxicity, easy to prepare, stronger stability, and high gelling capacities needing less quantities of gelator (<1 wt %) [5,6].

**Figure 1:**
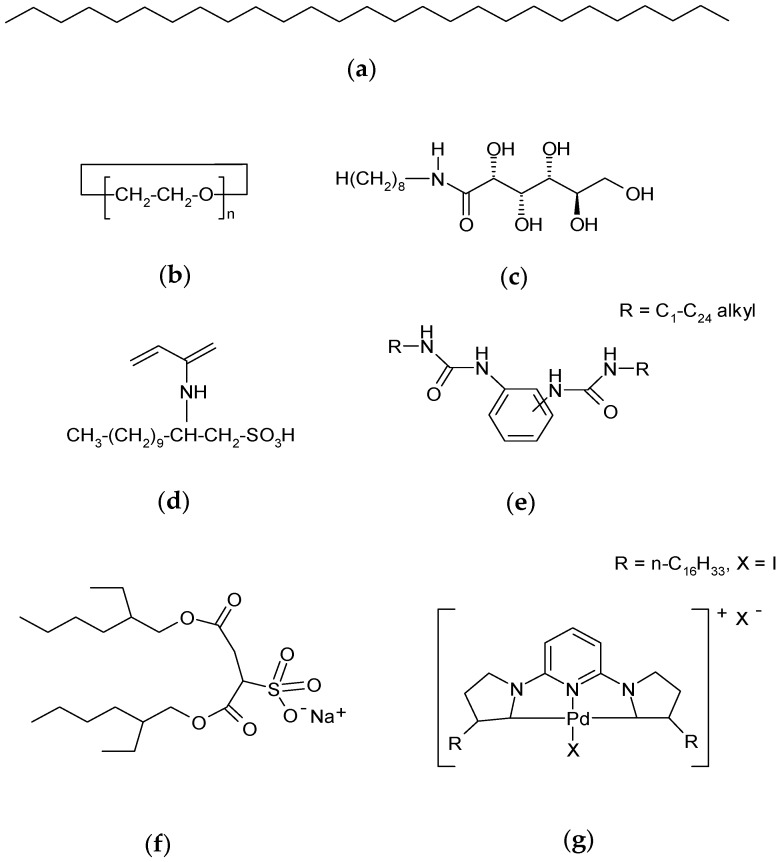
Examples of LMOG chemical structure: (**a**) n-octacosane; (**b**) 60-crown-20-macrocycle; (**c**) *N*-n-octyl-d-gluconamide; (**d**) 2-acryloylamide-dedecane-1-sulfonic acid (ADSA); (**e**) cyclic bis-urea gelator; (**f**) bis-(2-ethylhexyl) sodium sulfosuccinate (AOT); (**g**) palladium-CNC pincer bis(carbene).

The LMOG are separated into different classes according to criteria based on their molecular structure. For example, they can be classified as alkane organic gelators, as n-octacosane (Figure 1a), organic gelators with one heteroatom as ethers (e.g., 60-crown-20-macrocycle; Figure 1b), organic gelators with two heteroatoms (e.g., *N*-octyl-d-gluconamide; Figure 1c), organic gelators with three heteroatoms (e.g., racemic 2-acryloylamide-dedecane-1-sulfonic acid or ADSA; Figure 1d), polymerizable organic gelators such as bis-urea gelators (Figure 1e), two-component organic gelators such as bis-(2-ethylhexyl) sodium sulfosuccinate or AOT (Figure 1f), and organometallic gelators such as palladium-CNC pincer bis(carbene) (Figure 1g) [5,6].

**Figure 2:**
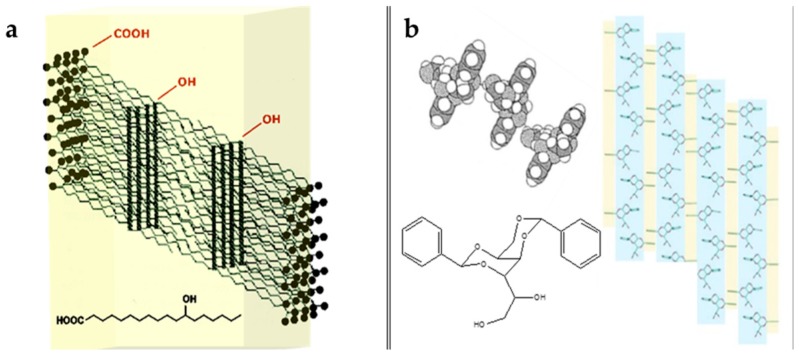
Chemical structure and representation of intermolecular interactions of: (**a**) HSA molecules; **(b**) DBS molecules.

Derived from this classification, several chemical compounds are used in cosmetics, such as 12-hydroxystearic acid (HSA) (Figure 2a), 1,3:2,4-di-*O*-benzylidene-d-sorbitol (DBS) (Figure 2b), sterols, lecithin, mono- and diglycerides, lecithin mixtures with sorbitan esters, fatty acids, fatty alcohols, waxes, and wax esters [5,6].

Plant metabolites—many of which are very structurally complicated—present attractive candidates as new LMOGs because they are available in renewable supply without extensive synthetic effort. Moreover, LMOG organogels can be classified into two groups depending on the kinetic properties of aggregates: “strong” organogel (solid fiber network) and “weak” organogel (fluid fiber network) [7,8,9].

In solution, LMOG self-assemble to form fibrous structures responsible for the gelation phenomenon [4]. Typically, the intermolecular forces that drive aggregation include hydrogen-bonding, π−π stacking, dipole−dipole, and London dispersion forces; however, hydrogen-bonding is a very important component of the self-assembly driving force in a large fraction of reported organogelators [10]. These physical gels are usually thermoreversible (reversible gel–sol/sol–gel phase transition by heating and cooling, respectively), depending on the molecular structure of the gelling agent and the fluid which is rigidified. It is possible to form nanoscale superstructures such as nanofibres, nanoribbons, nanosheets, nanoparticles, etc., which are of interest for materials and nanostructures conception. The interconversion between the gel and solution/sol phases depends only on the disassembly and assembly of the constituent molecules [7,11].

LMOGs are mostly used, since a limited number of synthetic polymers (e.g., polypeptides and polyesters) with these organogelation properties have been reported [12,13,14].

## 2. Preparation of LMOG Organogel Self-Assembled Systems

**Figure 3:**
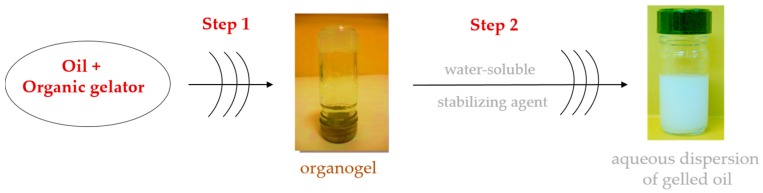
Schematic representation of gelled lipid nanoparticle (GLN) colloidal dispersion preparation process.

The formulation of stable colloidal dispersions of GLN includes two successive steps. The preparation of the organogel from an organic liquid (vegetable or emollient oil) and an organic gelator as the first step. After organogel preparation, aqueous dispersions of GLN are obtained by their dispersion in a water phase containing a stabilizing and/or emulsifying agent(s) [12] (Figure 3).

### 2.1. Gelation Process

**Figure 4:**
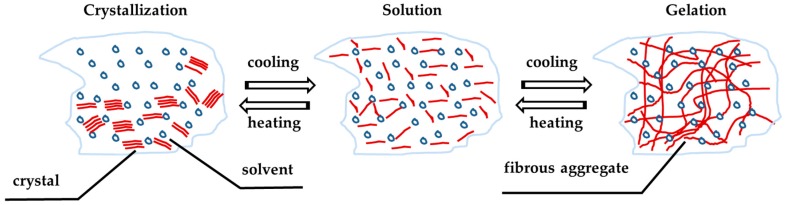
Organogel gelation–melting process representation.

LMOG organogels are obtained by dissolving a small amount of the organogelator at high temperature to obtain an isotropic solution and then cooling this solution under its characteristic gelation transition temperature, known as Tgel. The mixture must be completely solubilized and homogenized before being cooled below its Tgel, which represents the temperature from which the organogel formation process started. The Tgel has proven to be dependent on the concentration of the organogelator, the properties of the organic liquid (or oil), such as its polarity or viscosity, and in some cases of the cooling process. Gelation occurs as the hot solutions cools, while the self-assembled networks are formed. Since LMOGs form a physical gel (unlike chemical gels), these organogels are thermally reversible organic gel-like materials. The gelation process in this type of organogel is represented in Figure 4 [12].

It is important note that organogels formed by LMOGs tend to crystallize, leading to a more metastable organogel. It is possible to adhere a polymer as a gelation-driven compound with covalent bonds, converting it into a “polymer-based gelator” or supramolecular crosslinkable polymer organogelator that can form a more stable gel. For example, polysiloxanes, polyethers, and polycarbonates are useful polymers to confer a more stable characteristic to LMOGs [15]. Moreover, lecithin—a common organogelator usually used as stabilizing and dispersing agent to obtain stable organogel microemulsions—is expensive and difficult to obtain with high purity. When using lecithin of lesser purity, it can be used in the presence of synthetic polymers in the preparation of gel microemulsions in order to obtain the same stabilizing properties [16].

### 2.2. Phase Transition Parameters

The primary concept to consider in organogels is their phase transition parameters. These parameters are determined by rheology, as following the evolution of the elastic (G′) and viscous (G″) moduli versus temperature increase or decrease. The viscoelastic moduli permit the determination of the phase transition parameters of an organogel, such as gel–sol or sol–gel phase transition domains (PTDs), Tmelt (organogel melting temperature), Tgel, Tsol (organogel liquefaction temperature), as well as Tform (organogel formation temperature) [5,6].

**Figure 5:**
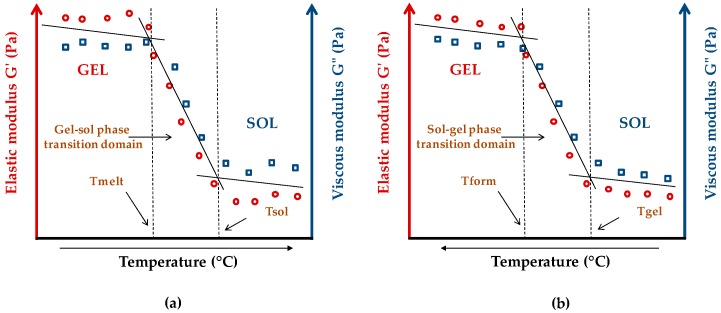
Schematic representation of phase transition parameters of an LMOG organogel: (**a**) organogel liquefaction process (temperature increase); (**b**) organogel formation process (temperature decrease).

Figure 5 shows the viscoelastic behavior of an LMOG organogel during its melting process (Figure 5a) and during its formation process (Figure 5b).

The rheological behavior of an LMOG organogel versus temperature presents two phases: the gel phase and the sol phase, separated by a transitional phase, known as PTD. According to the considered process, there is a gel–sol PTD (organogel liquefaction) or sol–gel PTD (organogel formation). The Tmelt corresponds to an abrupt decrease of the viscoelastic moduli and characterizes the beginning of the organogel melting process. This process is progressive, and takes place over a temperature range determined by the gel–sol PTD, and from a certain temperature (known as Tsol—organogel liquefaction), the organogel is completely liquefied. Concerning the sol–gel process (organogel formation), the Tgel corresponds to the abrupt increase of the viscoelastic moduli. Tgel indicates the beginning of the gelation process, contrary to the Tform which indicates the organogel formation temperature, above which the material shows constant viscoelastic properties with the temperature decrease. The sol–gel PTD is closed between Tgel and Tform.

The phase transition parameters of organogels obtained from HSA and various oils (emollients and vegetables) are reported in Table 1. The data show that Tgel and Tmelt values increase with increasing HSA proportion, regardless of the nature of the oil. Concerning Tsol and Tform, these parameters also increase when the HSA wt % increases and show higher values with vegetable oils, which is related to their important dynamic viscosity. The PTD range variation depends on the considered process. In the case of sol–gel PTD, it is closer than those of the gel–sol one. Moreover, this difference decreases as the proportions of HSA are increased for both types of oils (emollients and vegetable oils).

It is observed that when the proportion of HSA is increased, the sol–gel PTD increases and the gel–sol PTD decreases. The formation or destruction of the three-dimensional network of HSA fibers involves two very different processes. In the case of gelling, it is a highly cooperative process that is very energetically favorable. Moreover, during the melting process, there is a progressive breakdown of the network and the fluidization is complete only when all the HSA molecules are dissociated. These two processes are also very sensitive to the strength of the network junctions; that is, to their degrees of crystallization. The increase in HSA content increases the number of junctions in the network, which widens the sol–gel PTD because the network has a higher degree of interconnectivity. At low HSA wt %, a low density of junctions is observed, but these are strongly crystallized. Moreover, when the HSA content increases, the fiber network is more entangled, but the junction zones are more disordered and therefore break more rapidly during the gelling process.

### 2.3. Organogel Nanoparticle Dispersion

**Figure 6:**
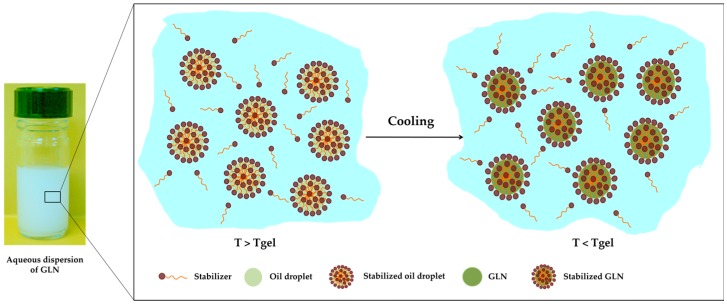
Schematic representation of GLN dispersion.

Tgel has an important part in the preparation of the GLN dispersions from a LMOG. As observed in Figure 6, the formation of these one depends on this parameter.

**Figure 7:**
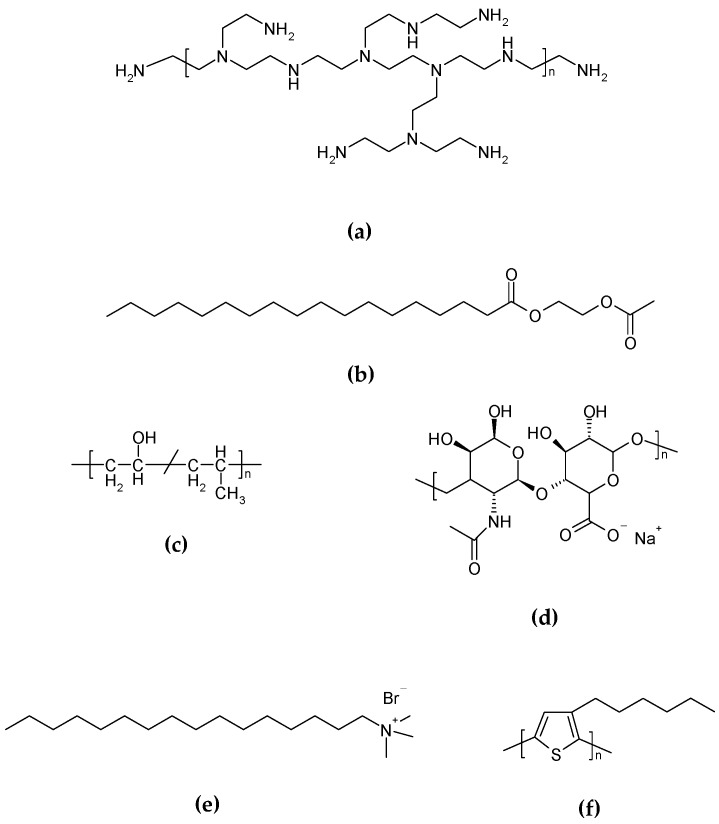
Chemical structure of common stabilizing agents used to prepare colloidal dispersions of GLN: (**a**) PEI; (**b**) AGS; (**c**) PVA; (**d**) SH; (**e**) CTAB; (**f**) P3HT.

The mixture must be heated over the characteristic organogel Tgel, and then homogenized while still hot by ultrasound probe or using mechanical homogenization in the presence of emulsifying, thickening, or stabilizing agents. For example, polyethylenenimine (PEI) (Figure 7a) [17], acetylated glycol stearate (AGS) (Figure 7b), and polyvinyl alcohol (PVA) (Figure 7c) are used as stabilizing agents, and sodium hyaluronate (SH) as a thickening one (Figure 7d) [4]. Another common surfactant used as a stabilizing agent for these preparations is cetyltrimethylammonium bromide (CTAB) (Figure 7e) [12]. Even though both homogenization methods have been reported, sonication with ultrasound probe has proven to produce a more stable aqueous dispersion obtained from vegetable oils and HSA as organogelator [4], as well as for organogel dispersions with poly(3-hexylthiophene) (P3HT) (Figure 7f) [18].

## 3. GLN Physicochemical Characterization

The organogel dispersions of GLN possess characteristic physicochemical properties. The organogel components and preparation process influence the physicochemical properties of its dispersion, such as particle size and distribution, and ζ-potential, morphology, and physical stability [4]. As for the preparation of the organogel dispersions, it is of great importance to determine GLN phase transition parameters based on rheological measurements and to be compared to the “parent” organogel [12]. It is important to perform a proper characterization of GLN properties in order to assess their physical stability or aging and potential applicability.

### 3.1. Influence of Ingredient Amount and Nature on the Physical Stability of GLN Aqueous Dispersion

The physical stability of GLN dispersions is of great importance for various applications, and it is essential to control particle destructive/unstable processes and monitor their stability. Parameters such as type and concentration of surfactant and oil as well as viscosity of dispersed phase play an important role in the physical stability of the dispersion, and have an impact on particle size [12]. For example, a dispersion of vegetable oil with a greater viscosity (60 cP) presents a better stability compared to a dispersion of mineral oil (6 cP). The viscosity must be enough to maintain the emulsifying and stabilizing agent on the gelled particle surface, allowing the system to have an adequate physical stability [4]. This stability is also maintained in the GLN aqueous dispersion, due to the high electrostatic repulsion between the particles due to the stabilizing agent anchored in the particle’s surface. [12].

While the concentration the organogelator has no influence on the particle size or on the physical stability, it has an effect on the viscosity of dispersions at high oil concentration. As described with other colloidal dispersions, particle size can be influenced by the formulation process parameters, such as shear speed during emulsification [12]. Dispersion can also be influenced by the pH, since the mean hydrodynamic diameter of a monodisperse population characterized by a high positive ζ-potential decreases with pH [2]. Some other parameters, such as the colloid size and surface area-to-volume ratio must be considered, and could have a strong influence for their potential uses.

### 3.2. Particle Mean Size, Distribution, and ζ-Potential

The light scattering method measures the time-dependent fluctuations of scattered light. Consequently, dynamic light scattering (DLS) provides information regarding the movement of the GLN in the colloidal dispersion, and uses Brownian motion to measure the mean hydrodynamic diameter and the size distribution of the particles. While smaller particles move faster, larger particles move slower, and the intensity of the fluctuations allows the particle size to be determined. Moreover, this method also permits the determination of the ζ-potential from the dynamic electrophoretic mobility. DSL studies show that by emulsifying bulk P3HT organogels into water-containing surfactant, two populations of particles are formed and there is an increase in size as a function of the P3HT concentration [18]. As for organogel dispersions obtain with HSA as gelator, the ζ-potential, polydispersity index (PDI), and particle size proved the influence of the concentration of the gelator on the stability of the dispersion [4]. Inclusive organogel dispersions made with dicarprylyl ether oil, HSA, and CTAB produced a stable colloidal system for longer than 45 days with GLN showing a mean diameter of around 260 nm, narrow PDI (0.01–0.1), and ζ-potential values varying between +25 and +50 mV [12].

### 3.3. Rheological Measurements

The rheological properties of organogel systems are of great importance with regards to preparing an aqueous dispersion of GLN and consequently to handling their properties and improve their potential applications. As described previously, gel–sol phase transition parameters of organogels are measured by following the variation of the elastic modulus (G′) and viscous modulus (G″) at increasing temperatures. The gelation process occurs as indicated by an abrupt increase of both moduli, while Tmelt is the exactly the opposite. When dispersed, the organogel showed similar rheological results, allowing the particle gelled state to be confirmed by demonstrating the existence of the Tmelt, Tsol, and gel–sol PTD [12].

**Figure 8:**
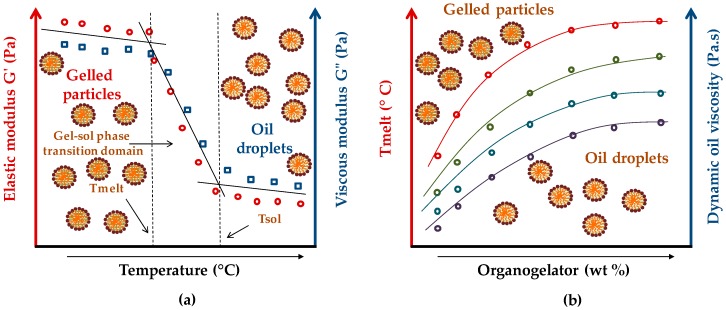
Gel–sol phase transition parameter evolution of GLN dispersion: (**a**) Tmelt, Tsol, and gel–sol PTD value determination according to the temperature increase; (**b**) Tmelt value evolution according the organogelator wt % and the dynamic oil viscosity increase.

Additionally, regarding the physicochemical properties of the organogels, colloidal dispersions of GLN possess some specific characteristics, giving them a broad spectrum of applications. In Figure 8a are reported the Tmelt, Tsol, and gel–sol PTD or phase transition parameters of gelled particles obtained from a vegetable (soybean, castor) or emollient (dicaprylyl ether, capric/caprylic triglycerides) oil, HSA as organic gelator, and PVA as stabilizing agent [12]. As previously noted, they are determined by rheology, measuring the evolution of the elastic (G′) and viscous (G″) moduli versus temperature. Tmelt is defined as the temperature below which gelled particles start to melt, while Tsol is the temperature from which the particles are totally transformed into oil droplets. The melting process is progressive, and takes place over a temperature gap, known as gel–sol PTD. Typically, Tmelt transition temperature increases with the concentration of organogelator and the viscosity of the oil used (Figure 8b) [4].

Moreover, the Tmelt is easily influenced by the type organogelator and its concentration. POG gels have a lower Tmelt and higher gel strength compared to organogels obtained using LMOGs [5,6].

The formulation of the dispersed organogel had an impact on their rheological properties. An increase of viscosity was observed in the ratio gelator/solvents, as it was observed for lecithin/isopropyl myristate microemulsions [19]. In the case of HSA, the same phenomenon has been observed. Measuring the flow behavior of aqueous dispersions of GLN, there was an increase of the organogelator concentration from 0 wt % to 15 wt %, leading to an increase in shear viscosity from two to six orders of magnitude [20]. The characteristic rheological properties of organogel dispersions are strongly influenced by the interactions between the GLN. The gelled particle dispersions showed a non-Newtonian behavior when the shear rate increased, leading to a decrease of the system dynamic viscosity values [4]. In contrast, in the case of a polymer-based organogel dispersion, the same effect was observed when diluting the dispersion from 40 vol % to 20 vol %, but the system showed a constant viscosity when diluted at 10 vol % because the interactions between the particles became negligible [18].

### 3.4. GLN Morphology

**Figure 9:**
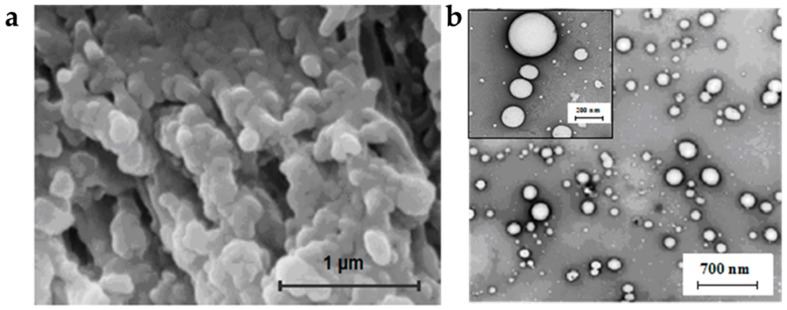
Electron microscopy pictures of GLN dispersion [4]: (**a**) SEM micrograph; (**b**) TEM micrograph. Copyright Clearance Center’s RightsLink^®^ service.

To evaluate the morphology and structure of the aqueous GLN dispersions, transmission electron microscopy (TEM) and scanning electron microscopy (SEM) were used, respectively (Figure 9).

These methods allow the data obtained from the DLS analysis to be completed. The DLS measurements provide insight into the size distribution of the gel particles within the dispersion, but they do not reveal information about their internal structure. In some applications, it is important that the particles retain porosity and a network structure original to the bulk organogel [18], while other GLN show spherical morphology without complementary information concerning their surface state [4]. These methods permit the accumulation of surfactant molecules at the surface of the gelled particles, confirming the ζ-potential values and indicating the stability of the dispersion [12].

## 4. GLN Potential Applications

Organogel dispersions of GLN are a suitable formulation for the preparation of new systems which could be used for cosmetic applications or as smart nanoparticles for drug delivery [4,12]. According to the data base, one of the first patent applications where the term organogel appeared was deposited organic solvent [21]. However, it was not until 1996 that the organogel dispersion concept appeared in European application patents. Notably, in the dermo-cosmetic field, companies like L’Oréal have patented dispersions of gel particles in a stabilized aqueous phase. [22].

Besides the cosmetic industry, organogel dispersions have been used as drug delivery systems and have proved their ability to enhance the permeation and improve drug release in dermal and transdermal formulations (Figure 9). It has been shown that organogel dispersions are effective in the improvement of sun protection factor (SPF) value, increasing the ability to absorb UV rays. This could be related to the optical properties of the particles and diffusing capacity provided by the organogelator. There is a synergistic improvement of two factors: the photo-protective ability and the photo-stability of the UVB filter [4]. Moreover, it has been used for topical treatments of early stages of skin cancers. In addition, these systems could be used for intravenous applications by adapting the formulation for this aim [3].

### 4.1. Hydrophobic Reservoirs for Sun Protection

**Figure 10:**
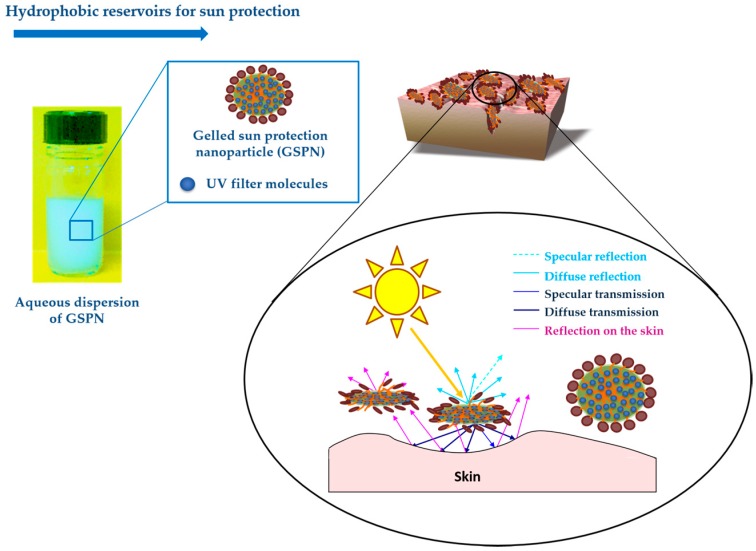
Representation of gelled sun protection nanoparticles (GSPN) and their photoprotection ability mechanism.

Gelled sun protection nanoparticles (GSPN) in water could be employed as a nanocarrier for dermo-cosmetic applications or as hydrophobic reservoirs for photoprotection (Figure 10) [4,5,6,17,23].

**Figure 11:**
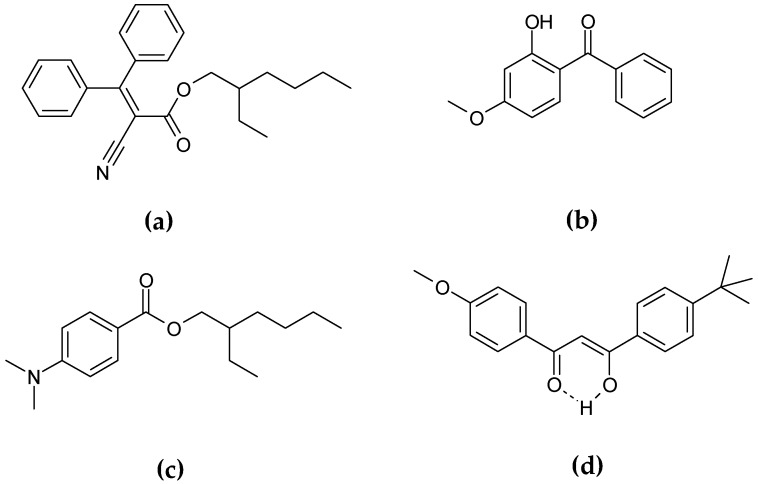
Chemical structure of immobilized chemical UV filter as GSPN: (**a**) octocrylene; (**b**) benzophenone-3 (BP-3); (**c**) 2-ethylhexyl-p-dimethylaminobenzoate (EHMAB); (**d**) avobenzone.

The preparation process and physicochemical properties of these GSPN aqueous dispersions have been described previously by Chaouat et al. [24]. Gelled particle dispersions of sunscreen organic mixture–1-(4-methoxyphenyl)-3-(4-*tert*-butylphenyl)propane-1,3-dione or avobenzone (UVA solar filter) (Figure 11a) dissolved in 2-ethylhexyl 2-cyano-3,3-diphenyl-2-propenoate or octocrylene (UVB solar filter, viscous liquid) (Figure 11b) and HSA as organic gelator were obtained by emulsification at high temperature and cooling at room temperature, using PVA as a stabilizing and dispersing agent. The results confirmed that the GSPN size was in agreement with the necessity of keeping the particles on the skin surface, avoiding their penetration through the *stratum corneum*. TEM observation of the dispersion showed spherical particles with a mean diameter of 600 nm—a size in accordance with the DLS measurements. The UV spectroscopy observations showed more important absorption of the gelled particles compared to that of corresponding emulsion droplets. A rheological investigation allowed the particle sol-gel phase transition parameters to be determined, confirming their gelled state. A comparative aging study of the emulsion and corresponding dispersion showed greatly enhanced physical stability after gelation. This last point was also proved by ζ-potential measurements.

According to Kirilov et al., aqueous dispersions of GSPN were also obtained with two organic liquids [4]. Vaseline and almond oils were used as organic media for the GSPN preparation. Gelled nanoparticles containing a model sunscreen molecule with mean size of 450 nm, ζ-potential value above −30 mV, and PDI of 0.18 were obtained by the ultrasound probe homogenization method. A comparative study of their dispersion aging showed a greatly enhanced GSPN stability after gelation. According to the UVB absorption evaluation, GSPN improved the photoprotective ability and the photostability of the immobilized UVB blocker as 2-ethylhexyl-p-dimethylaminobenzoate or EHMAB (Figure 11c). In addition, GSPN showed a high water resistance (~83%), even after 40 min of immersion.

Actually, studies concerning the immobilization of benzophenone-3 or BP-3—a commonly used UVA and UVB absorbed molecule—as GSPN are being carried out (Figure 11d).

The obtained results demonstrate the interest in GSPN and their aqueous dispersion as a concept for the preparation of original sun protection formulations with improved diffusion properties and waterproof ability.

### 4.2. Hydrophobic Reservoirs for Drug Delivery

**Figure 12:**
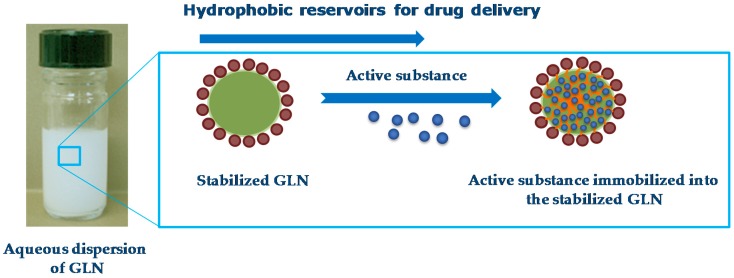
Representation of the drug delivery ability of GLN.

Other studies demonstrated the interest of colloidal dispersions of GLN for the encapsulation and delivery of lipophilic active compounds (Figure 12).

**Figure 13:**
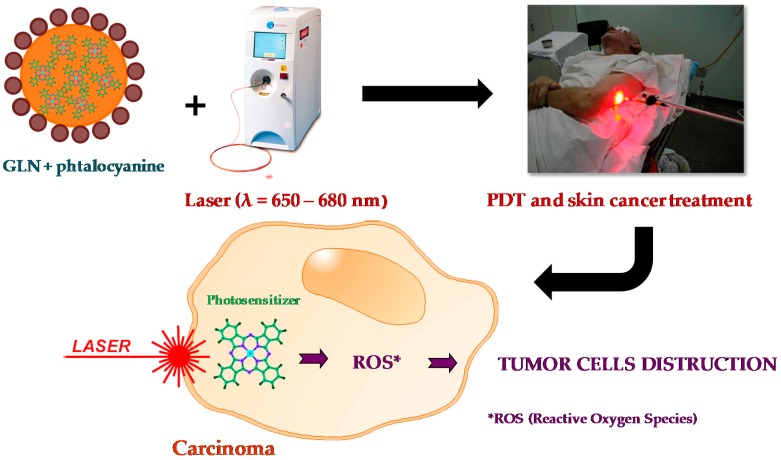
Representation of the medical treatment of skin cancers using photodynamic therapy (PDT).

Sigueira-Mourra et al. prepared stable dispersions of soybean oil GLN with excellent chloroaluminum phthalocyanine (ClAlPc) encapsulation efficiency. The obtained ClAlPc-loaded GLN formulation could be a very useful system for photodynamic therapy (PDT) procedures (Figure 13).

**Figure 14:**
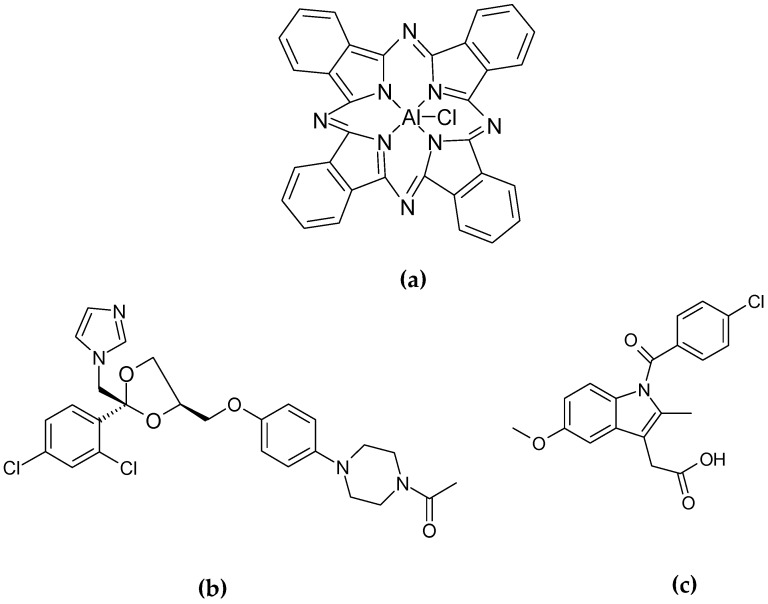
Chemical structure of immobilized active substances: (**a**) chloroaluminum phthalocyanine (ClAlPc); **(b**) ketoconazole; (**c**) indomethacin.

Authors have efficiently encapsulated ClAlPc (Figure 14a)—a second-generation photosensitizer agent usually used in PDT—into soybean oil GLN dispersed in aqueous medium. Nanoparticles were jellified and stabilized by HSA and PEI, respectively. They were obtained by hot homogenization using ultrasound probe and then cooling at room temperature until obtaining a stable GLN dispersion. Nanoparticles showed mean diameter of 280 nm, narrow size distribution (PDI ~ 0.3), positive ζ potential value (~+50 mV), and spherical morphology (Table 2). Dispersions exhibited pronounced physical stability after a 6-month aging period. The PEI and HSA proportions allowed the GLN size, charge, and stability to be controlled, resulting from the complex electrostatic interactions between the positively-charged PEI and the negatively-charged HSA fibers present on the gelled particles’ surface on the one hand and stearic repulsions between positively-charged GLN on the other hand.

Martin et al. studied the release profile and efficiency of GLN obtained by the same preparation process as Siqueira-Mourra et al. [24]. In the first step, they used DLS and differential scanning calorimetry (DSC) to select the most suitable formulations by optimizing the proportion of ingredients (HSA, PVA, castor oil) to obtain particles of the smallest size and greatest stability. Two lipophilic drug models—indomethacin (Figure 14b) and ketoconazole (Figure 14c)—were entrapped in the GLS made of castor oil and gelled by HSA.

Thermal studies confirmed that there was no significant alteration of gelation phenomenon in presence of the entrapped drugs. Very stable dispersions were obtained (>3 months), with GLN presenting a mean diameter between 240 and 300 nm. High encapsulation efficiency (>98%) was measured for indomethacin and ketoconazole (Table 2). The release profile determined by in vitro dialysis showed an immediate release of the drug from the organogel nanoparticles, due to rapid diffusion.

All these studies demonstrate the interest in the colloidal dispersions of GLN for dermo-cosmetic applications as sun protection systems and for drug delivery for lipophilic active substances.

## 5. Conclusions

Since lipophilic drug formulation remains the major challenge in the pharmaceutical field, recent preparations of colloidal dispersions of GLN present very interesting advantages as drug delivery formulations as well as in the cosmetic industry. These dispersions have promising stability properties, are easily formulated, and can be produced with natural organogelators or simple synthetic polymers, giving them a great range of possible industrial applications. The basic knowledge of these colloidal GLN dispersions, their properties, and characterization methods will allow the optimization of the formulation with regards to obtaining more stable dispersions according to their intended applications. Furthermore, they are still a broad area of possibilities in the synthesis of new organogelators, formulations of better and more stable dispersions, and drug immobilization. The interest for these materials will continue to grow in the future years.

## Figures and Tables

**Table 1:** Phase transition parameters of organogels obtained from emollients (dicaprylyl ether, capric/caprylic triglycerides) or vegetable oils (soybean, safflower) and 3, 6, and 15 wt % of HSA, respectively. PDT: phase transition domain.

Emollients or vegetable oils	Tgel ^1^ (°C)	Tform ^1^ (°C)	Sol–Gel PTD (ΔT)	Tmelt ^1^ (°C)	Tsol ^1^ (°C)	Gel–Sol PTD (ΔT)
**Oil/HSA 3 wt %**						
ether	49	45.5	3.5	48	63	15
triglycerides	47	44	3	48.5	61	12.5
soybean	51	47.5	3.5	53	67	14
safflower	54	50	4	53	68	15
**Oil/HSA 6 wt %**						
ether	56	51.5	4.5	55	65	10
triglycerides	56	51	5	54	64	10
soybean	58	53.5	4.5	58.5	68	9.5
safflower	61	56	5	59	70	11
**Oil/HSA 15 wt %**						
ether	63	56	7	62	71	9
triglycerides	63.5	55.5	8	61	70	9
soybean	66.5	60	6.5	66	71	5
safflower	65	56	9	63	72	9

^1^ T ± 0.5 °C.

**Table 2:** Some experimental results from physicochemical characterization GLN containing active substances (*n* = 3), pH = 7. PDI: polydispersity index.

Active Substance	Mean Size (nm)	PDI	ζ-Potential (mV)	Encapsulation Efficiency (%)
ClAlPc	280 (±10)	0.3 (±0.04)	+50 (±1.6)	~100
indomethacin	244 (±13)	0.26 (±0.05)	−30 (±5.6)	91.9 (±0.2)
ketoconazole	266 (±16)	0.25 (±0.03)	−25.9 (±4.7)	97.4 (±0.1)
